# The Frontal Eye Fields Limit the Capacity of Visual Short-Term Memory in Rhesus Monkeys

**DOI:** 10.1371/journal.pone.0059606

**Published:** 2013-03-15

**Authors:** Kyoung-Min Lee, Kyung-Ha Ahn

**Affiliations:** Department of Neurology, Seoul National University Hospital, Seoul, Republic of Korea; University of Montreal, Canada

## Abstract

The frontal eye fields (FEF) in rhesus monkeys have been implicated in visual short-term memory (VSTM) as well as control of visual attention. Here we examined the importance of the area in the VSTM capacity and the relationship between VSTM and attention, using the chemical inactivation technique and multi-target saccade tasks with or without the need of target-location memory. During FEF inactivation, serial saccades to targets defined by color contrast were unaffected, but saccades relying on short-term memory were impaired when the target count was at the capacity limit of VSTM. The memory impairment was specific to the FEF-coded retinotopic locations, and subject to competition among targets distributed across visual fields. These results together suggest that the FEF plays a crucial role during the entry of information into VSTM, by enabling attention deployment on targets to be remembered. In this view, the memory capacity results from the limited availability of attentional resources provided by FEF: The FEF can concurrently maintain only a limited number of activations to register the targets into memory. When lesions render part of the area unavailable for activation, the number would decrease, further reducing the capacity of VSTM.

## Introduction

Primates process visual information with remarkable efficiency, yet can hold only a limited number of discrete locations or objects in memory at one time [1]–[3]. This limit is easily demonstrable, for instance, by situations evoking change blindness, i.e., the failure to detect obvious differences between images when separated by time or space, and by neuropsychological tests probing the visual short-term memory (VSTM). The capacity limit in VSTM has been estimated to be three or four items in both human [4]–[8] and non-human primates [2], [9], [10].

The frontal eye fields (FEF) seems to play an important role in VSTM [11]. A majority of neurons in the area respond to visual events and visual responses in some outlast the stimulus [12], potentially subserving the short-term memory. In fact, when the FEF is temporarily inactivated by chemicals such as muscimol, a GABA agonist, or lidocaine, a local anesthetic, memory-guided saccades are impaired: Saccades directed to a briefly flashed target can no longer made, whereas those to a visible target are much less affected [13]–[16]. However, many details still remain unanswered regarding the nature of short-term memory carried out by FEF, and in this study we addressed the following two specific questions.

First, we asked whether or not FEF inactivation would affect the VSTM capacity. Recent works demonstrated the bilateral advantage in visual tracking [17] and visual working memory [18]: Bilateral presentations of visual stimuli lead to an increased probability of storage in memory and better performance in tracking than unilateral presentations. The bilateral advantage implies that these functions are carried out in the left and right hemifields independently, imposing a constraint on the potential neural substrates for the functions [19]. Since the FEF surely meets this requirement of hemifield independence, we asked whether rendering FEF unavailable for activation would reduce the behavioral capacity of VSTM.

Second, numerous studies have implicated the FEF in attention shift [20]–[25], and we wondered how the short-term memory function by this area would relate to the deployment of spatial attention by the same area. We designed task conditions where the distribution of saccade targets and the bottom-up visual attention prompted by the targets would vary between contralesional and ipsilesional sides during memory encoding. Analysis of memory-guided saccades as a function of target distribution indeed revealed an interaction between visual attention and the effect of FEF inactivation on VSTM. The interaction, consistent with the retinotopic mapping in FEF, was observable only when the two functions are taxed with multiple targets.

## Materials and Methods

### Ethics Statement

All experimental procedures were approved by the Seoul National University Hospital Animal Care and Use Committee (IACUC No: 09-0166, Project Title: Neural mechanisms of saccade choice in primate frontal cortex), and followed the US Public Health Service Policy on the humane care and use of laboratory animals. All animals used in this study were cared for at a temperature- and humidity-controlled room in the Primate Center of Seoul National University Hospital. While they were housed in individual cages, social contacts were encouraged by regularly opening a retractable door between cages. Environmental enrichment was also provided with a variety of toys. The animals were provided with a regular chow for monkeys, supplemented by fresh fruits. The health status of the animals was monitored daily by care-givers and by regular physical examinations and blood tests by the staff veterinarian. At the end of experiments, each animal was euthanized by deep anesthesia with zoletil chloride (10 mg/kg IM) and sodium pentobarbital (100 mg/kg, IV). After confirming total lack of a corneal reflex as an indication of adequate level of anesthesia, the animal was perfused with a liter of 0.1% phosphate buffered saline followed by several liters of fixative solution (10% buffered formalin). This procedure is consistent with the recommendations of the Panel on Euthanasia of the American Veterinary Medical Association [26].

### Subjects and Surgical Preparation

Two adult female rhesus monkeys (*Macaca mulatta,* M9 and M10) weighing between 4 and 5 kg were prepared for chemical inactivation experiments. They were the same animals previously used in another study [27]. A head-restraint post and recording cylinders were implanted under isoflurane anesthesia and sterile surgical conditions. The recording cylinders (20 mm, internal diameter) were positioned over craniotomies centered on the right arcuate sulcus in all animals.

### Procedures to Minimize Animal Discomfort, Distress, Pain and Injury

Three situations existed in which a monkey might experience discomfort, distress and/or pain in our experimental protocols: a) survival surgery; b) restraint for handling or routine testing and c) training and experimental recording sessions. The following steps were taken to ameliorate animal suffering in each situation. **a) Survival surgery.** The purpose of the surgical procedures was to implant recording chambers and a head restraint device for neurophysiological experiments. All surgeries were carried out in the animal surgical suite at the Primate Center of Seoul National University Hospital. Animals were prepared with sterile, anesthetic surgical procedures. A licensed veterinarian was present throughout the surgical procedures and the recovery period for anesthetic induction and for monitoring and recording all measured physiological variables. Animals were allowed free access to water but no food the night prior to scheduled surgery. One hour before the surgery the animal was given atropine sulfate (0.08 mg/kg, IM) to prevent excessive salivation during the surgery. One-half hour later it was sedated with zoletil chloride (10 mg/kg, IM), intubated, and placed under isoflurane anesthesia. A saline drip was maintained through an intravenous catheter placed into a leg vein. Throughout the surgery, core body temperature, heart rate, blood pressure, oxygen saturation and respiratory rate was continuously monitored. The animal was returned to its home cage after waking from the anesthesia and allowed to recover fully from the effects of surgery before behavioral training started. During the period of post-surgical recovery the animal was monitored closely and given injections of an analgesic agent (meloxicam 0.4 mg/kg IM) and antibiotics (cephazolin, 25/mg/kg IM) in consultation with the veterinarian for 3 days post-op. **b) Restraint for handling or routine testing.** Restraint for certain procedures, such as physical examination or blood sampling for health check, was accomplished with zoletil chloride (10 mg/kg IM). **c) Training and experimental recording sessions.** After recovery from the surgical procedure the animal was trained to be held by the arms and moved into a large plastic primate chair. This was done by supplying the animal with rewards of fruit and juice. The chair had a perch with an adjustable height for each animal’s comfort. Wastes fell into a collection pan below the animal, and thus, did not cause the animal discomfort. The animals were trained by the delivery of water or fruit juice rewards in daily sessions during which time they received their entire liquid intake in the experimental apparatus. When the animal was fully trained the experiments began. During the experimental sessions the animal’s head was painlessly restrained through the use of the implanted head post which mated to a vertical rod attached to the primate chair. The animals did not show any sign of discomfort by the head restraint device: They continued to train steadily for the period of time that they were in restraint and often fell asleep as they sat in the darkened room between blocks of trials.

### Behavioral Tasks

#### 1) The multi-target memory-guided saccade task (MEM, Figure 1A, upper panel)

A trial began with the appearance of a white 1.0-degree square at the center of visual field. Four hundred milliseconds after the animal started fixation at the square, a four-by-three matrix of circular discs was presented. The color of discs distinguished targets (red) from non-targets (green), and the animal was trained to make saccades to the targets. Now, as soon as the first saccade was initiated, the targets were rendered green and indistinguishable from non-targets. The animal therefore had to rely on memory in order to make subsequent saccades to the second target and onwards. A drop of water reward was given after all targets were visited by at least one saccade.

The location of targets varied randomly across trials, but the target number, ranging from one to four, remained constant in a block of 40 trials. Blocks were randomly ordered and counter-balanced in terms of the target number, so that blocks of the same target number were run twice in a session.

Each disc was 1.0 degree in diameter with luminance of 124 cd/m^2^ with the black background of 1.70 cd/m^2^ (measured by a chromameter, CS-100; Minolta Photo Imaging, Mahwah, NJ). The rows and columns of discs were separated by 15 degrees, such that the matrix covered a visual field of 45 degrees horizontally and 30 degrees vertically.

The purpose of using the matrix-form arrangement of target locations, instead of a more traditional circular array, was to explore a wider range of visual fields with targets evenly spaced. In the circular array, the vector relationship between the first and the second saccades is biased to roughly opposite directions. In contrast, the matrix formation has the advantage that the target array remains similar for successive saccades in a series. For instance, after the first saccade to the nearest left or right target from the central fixation, a similar target array becomes available for the second saccade. This will result in a more even mixture of contra- and ipsilesional saccades in series. Thus, the matrix array would be better in general for studying saccade sequences such as in saccade remapping. By the same token, it would also be useful in examining the eye-position effect on visual or oculomotor functions, for instance, with respect to eye- versus head-centered coordinates.

The task for the animal was to make at least one saccade to each target, and water reward was delivered as soon as all targets had been visited. The order of visits was left up to the animal. Repeated saccades to the same target was permitted, as was saccades to non-targets. Constraints were set on the response period such that a trial would be terminated if the total number of saccades exceeded two times that of targets (i.e., two saccades in trials with a single target, four with two targets, etc.) or the total elapsed time after the matrix onset surpassed 400 ms times the target number (for instance, 400 ms in single-target trials and 1600 ms in trials with four targets). However, once trained, the animals rarely reached these constraints, and the saccades formed an efficient and specific trajectory, stopping only on targets and once at each target.

Given these behavioral requirements, the chance levels in the memory task were calculated using a bootstrap method: Serial saccades to twelve target locations were numerically simulated using MATLAB (The Mathworks, Natick, MA, USA). Ten thousand blocks of forty sample-trials each were generated and trials were counted where the random series of saccades met the behavioral constraints for reward described above. According to this simulation, the chance levels of trial success rates are 16.6, 7.9, 5.7, and 5.1% for the target number of one through four, respectively, when the saccades were sequenced with the possibility of revisit to targets. When inhibition of return was imposed, the chance levels were higher at 16.6, 9.1, 9.0, and 14.1% for one to four targets, respectively.

#### 2) The multi-target visually-guided saccade task (VIS, Figure 1A, lower panel)

Events in this task were the same as in the MEM task above, except one feature that the red targets remained visible throughout the trial. Therefore, the animals did not need to rely on short-term memory to reach the second target and onwards. When there was only one target, VIS was identical to MEM.

VIS served as a baseline condition in comparison with MEM. In both tasks, saccade targets were selected based on salient color contrast. While VIS did not require memory of the targets, the planning and execution of sequential saccades was comparable with MEM. Other details, such as constraints on the response period and behavioral measures were the same in both.

### Eye Tracking Data Acquisition

Eye movements were monitored by infrared video-oculography with a sampling rate of 500 Hz (Eyelink2, SR Research Ltd, Kanata, Ontario, Canada). Events in the tasks were controlled and saccade behavior measured on-line by custom-made applications written in MATLAB (The Mathworks, Natick, MA, USA). Markers were set for all experimental events for off-line analysis. The onset and offset of saccades were determined by velocity criteria (30°/s radial velocity for onset and 10°/s for offset). This was performed on-line to control the events in the behavioral tasks, such as detecting the first saccade and hiding targets in MEM. Correct detection of saccade onsets was confirmed during off-line data analysis.

Given the matrix arrangement of the visual stimuli, saccades were judged as directed to the nearest disc, regardless of the absolute distance from the disc to the saccade end-point. In other words, a saccade was judged as directed to a disc if it ended within a square window of 15-degree width and height centered at the disc.

### Muscimol Inactivation

**Figure 1:**
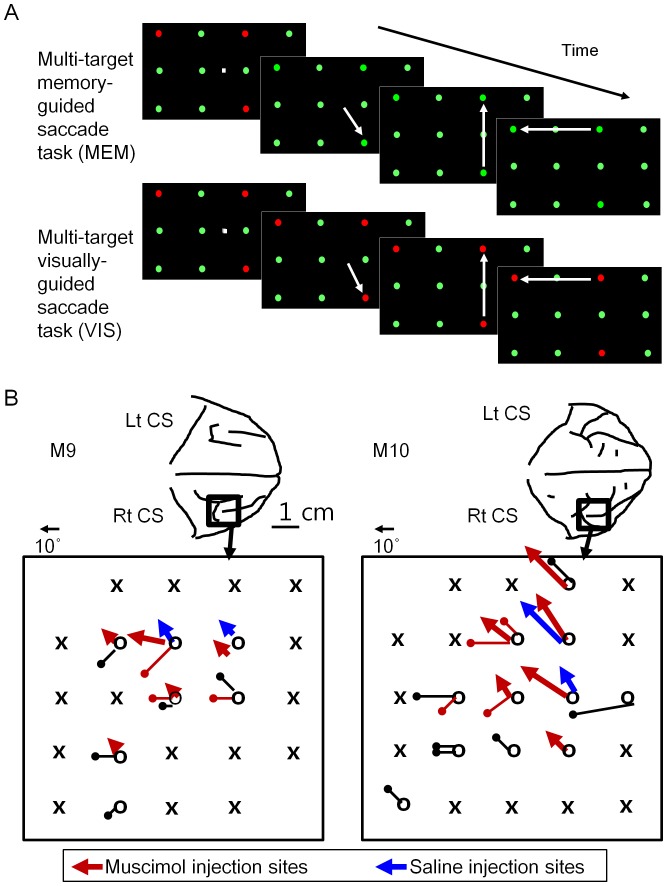
The behavioral tasks and the FEF inactivation sites are schematically depicted. (**A**) The multi-target visual short-term memory and pop-out search tasks. After fixation at the central square, a set of discs were shown in either red or green. The red discs were saccade targets defined by the color contrast. In the multi-target memory-guided saccade task (MEM, upper panel), the targets were rendered indistinguishable from non-targets as soon as the first saccade was initiated. The animal therefore had to rely on short-term memory for subsequent saccades. In the visually-guided saccade task (VIS, lower panel), the targets were visible while the animal made a series of saccades to each target, obviating the need for memory. (**B**) Sites in the FEF are shown where saccades were evoked by electrical stimulation (black circles). X’s denote sites with no saccade evoked with the current level up to 300 microampere, and colored lines indicate that the current threshold of saccade evocation was lower than or equal to 50 microampere. Red and blue arrowheads mark the penetrations with injection of muscimol (red) or normal saline (blue), whereas a circular head indicates sites that were tested but not infused. Each arrow coming from a circle represents the vector of saccades evoked at the site and depth of injections. Some sites were penetrated more than once, and the lines were slightly shifted for clarity.

To identify and map the FEF (Figure 1B), tungsten electrodes (FHC Co., USA) were introduced through a guide tube positioned by a grid system (Crist Instruments Co., USA). A cortical sites of electrode penetration was regarded as within the FEF if a saccade was evoked with a probability greater than 0.5 by electrical stimulation (ES) with a current less than or equal to 50 microampere (negative-first biphasic pulses with 0.1 ms in each phase, 100 Hz train frequency, and 200 ms train duration). For each track of penetration, the electrode was slowly lowered by a electrical microdrive (NAN Instruments Ltd, Nazareth, Israel) and responses to ES were checked at every 0.5 mm interval in depth. The depth with the lowest threshold of ES-evoked saccades was marked on the way into the cortex and confirmed again on the way out.

An injectrode constructed using a 33-gauge hypodermic cannula was inserted at the same site as the electrode, and lowered slowly until its tip was located at the depth marked as having the minimal ES threshold. Muscimol (or saline in control experiments) was injected using a minipump (Aladdin 1000, World Precision Instruments, Sarasota, FL, USA). The muscimol concentration was five mg/ml, and the injected volume was one microliter over about two minutes. Following the injection, the cannula was left in place for about five minutes before it was withdrawn. Data collection began immediately before the injection and continued up to three hours. Data were also collected in the following day, and full recovery was always noted. The injection experiments including the saline control were separated by at least two days.

In order to obtain homogenous behavioral effects, muscimol injections were made at FEF sites where ES-evoked saccades were directed to the left either horizontally or with an upward component and the current threshold was lower than or equal to 50 microampere (penetrations marked by red arrows shown in Figure 1B, five sites in M9, and six in M10). For control experiments, two sites in each monkey were tested by injecting saline (penetrations marked by blue arrows in Figure 1B).

### Behavioral Measures and Statistical Analysis

Two behavioral measures were assessed from the eye traces: (1) the trial success rate, or the proportion of trials rewarded, reflecting the overall performance in the tasks, and (2) the saccade proportion rate to each target location, i.e., the proportion of trials where at least one saccade was made to the location over trials in which a target was shown at that location. This measure was used to assess the spatial distribution of effects by FEF inactivation.

Two-way analysis-of-variance (ANOVA) was used to test statistical significance of the main effects of time after an injection and task type (i.e., with or without memory requirement) or target number. Interactions between post-injection time and task type or between post-injection time and target number were also tested. The Kruskal-Wallis test (a non-parametric one-way ANOVA) was used for comparisons over categorical variables. A threshold of p<0.05 was regarded as evidence of statistical significance, and the p values reported. All statistical analyses were conducted using MATLAB (Mathworks, Natick, MA).

## Results

### Performance in the MEM Task was Impaired during FEF Inactivation, but not in VIS

**Figure 2:**
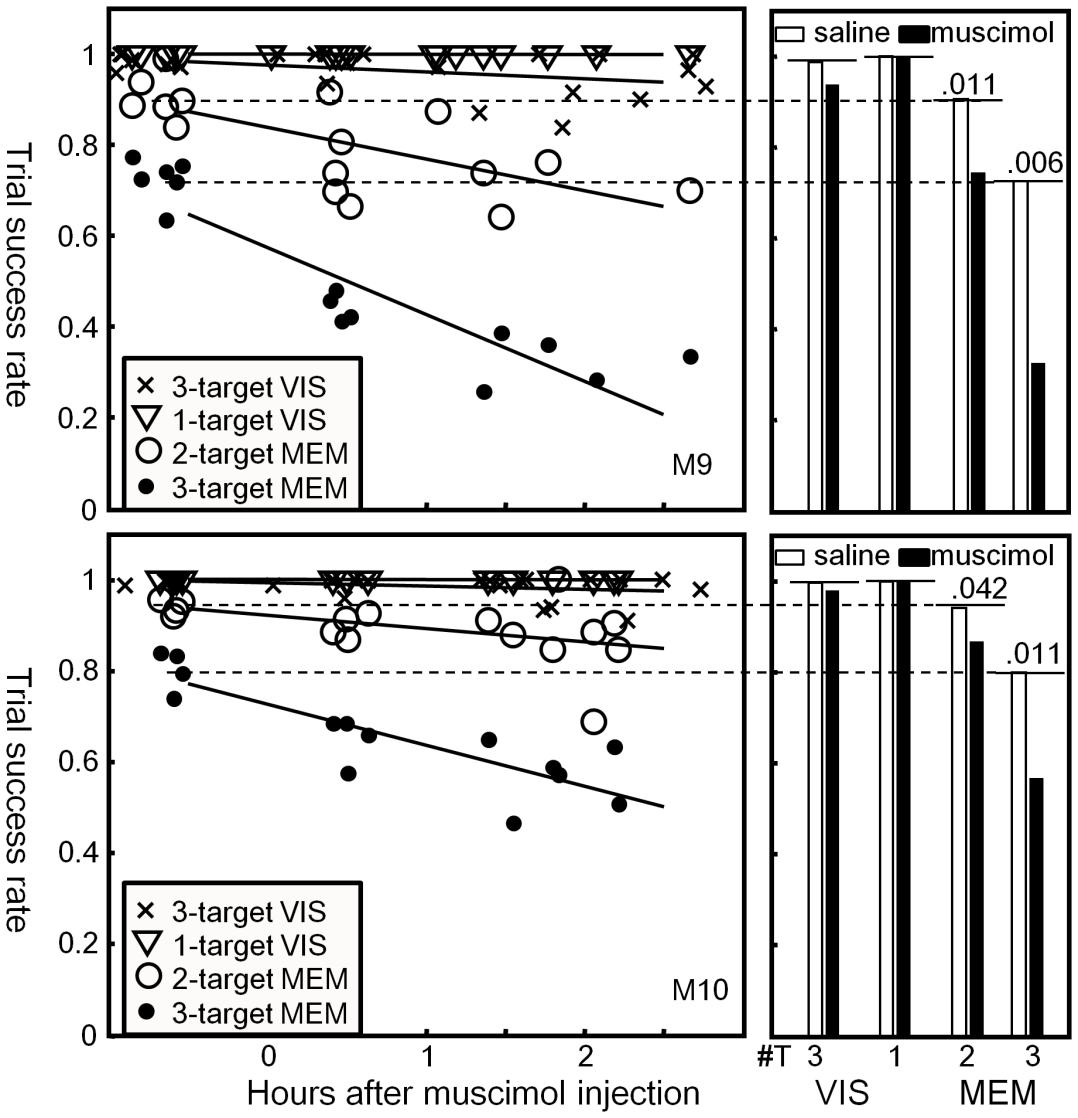
Effects of FEF inactivation on the overall performance of the multi-target memory- and visually-guided saccade tasks (MEM and VIS, respectively) are shown. The trial success rate, i.e., the ratio of rewarded trials over total trials, is plotted as a function of time after muscimol injection with regression lines. Shown are search conditions with one or three targets (▽ and x, respectively) and memory conditions with two or three targets (○ and •, respectively). Data were pooled over five and six experiments with M9 and M10, respectively. Location of the inactivated FEF sites is given in Figure 1B. The bar graphs on the right represent the TSR more than one hour after muscimol (black bars) or saline injections (white bars). A significant difference (p<0.05) by the Kruskal-Wallis test between the two injection types was indicated by the p value above the bars. Horizontal dashed lines indicate the pre-injection TSR levels. ^#^T: number of targets.

After a few months of training on the VIS and MEM tasks (Figure 1A), the performance of two rhesus monkeys (*Macaca mulatta,* M9 and M10) reached an asymptote. For both animals, the trial success rate (TSR) in four-target MEM was consistently lower than 50%. Since the animals often got frustrated with the low yield of reward and refused to work, MEM was tested only up to three targets in inactivation experiments. To use the reward sparingly, the VIS task was administered only with one or three targets, skipping the two-target condition. Mean TSR before FEF inactivation were: in VIS with one target and three targets, >99% for both animals; in MEM with two targets, 89% by M9 and 93% by M10; and in MEM with three targets, 67% by M9 and 79% by M10 (pre-injection data shown in Figure 2).

During reversible inactivation of the right FEF (sites with red arrows in Figure 1B) by muscimol, the performance in MEM was impaired (Figure 2): TSR declined over time following muscimol injection in the two- and three-target memory conditions (○ and •, respectively). The TSR data in MEM were tested using two-way ANOVA for two main factors: time after injection (a continuous variable) and number of targets per trial (NT, two versus three memory targets). Both main effects were significant as well as their interaction in both monkeys (two-way ANOVA, the time effect F = 62.7, p = 3.9×10^−7^, the NT effect F = 25.2, p = 4.2×10^−8^, the interaction F = 22.7, p = 1.4×10^−7^ for M9; time effect F = 29.9, p = 2.9×10^−6^, NT effect F = 12.3, p = 7.1×10^−5^, interaction F = 12.6, p = 5.9×10^−5^ for M10).

In contrast, the performance in VIS was unaffected by FEF inactivation. For both animals, the TSR in the one- and three-target search tasks (▽ and x in Figure 2, respectively) stayed close to one with no significant change after injection, regardless of target number (two-way ANOVA, time effect F = 2.8, p = 0.10, NT effect between one and three targets F = 0.3, p = 0.57, interaction F = 2.8, p = 0.10 for M9; time effect F = 1.4, p = 0.24, NT effect F = 0.05, p = 0.83, interaction F = 1.4, p = 0.24 for M10).

A direct comparison between the three-target MEM and VIS tasks confirmed the task effect (two-way ANOVA, time effect F = 70.1, p = 7.1×10^−10^, task effect between MEM and VIS F = 51.4, p = 2.3×10^−8^, interaction F = 47.0, p = 5.9×10^−8^ for M9; time effect F = 58.6, p = 4.6×10^−7^, task effect F = 47.8, p = 4.3×10^−8^, interaction F = 43.5, p = 1.1×10^−7^ for M10).

The specificity of muscimol injection was demonstrated in comparison with saline injection (Bar graphs on the right side in Figure 2): The performance after saline injection remained at the same level as before the injection (not different from the pre-injection levels indicated by horizontal lines in the figure), while it declined after muscimol injections. TSR more than one hour after muscimol (black bars) and saline injections (white bars) were statistically significantly different in the two- and three-target MEM conditions in both monkeys (p values given in the figure by the Kruskal-Wallis test).

### The Impairment in MEM was not Attributable to Changes in Saccade Behavior

**Figure 3:**
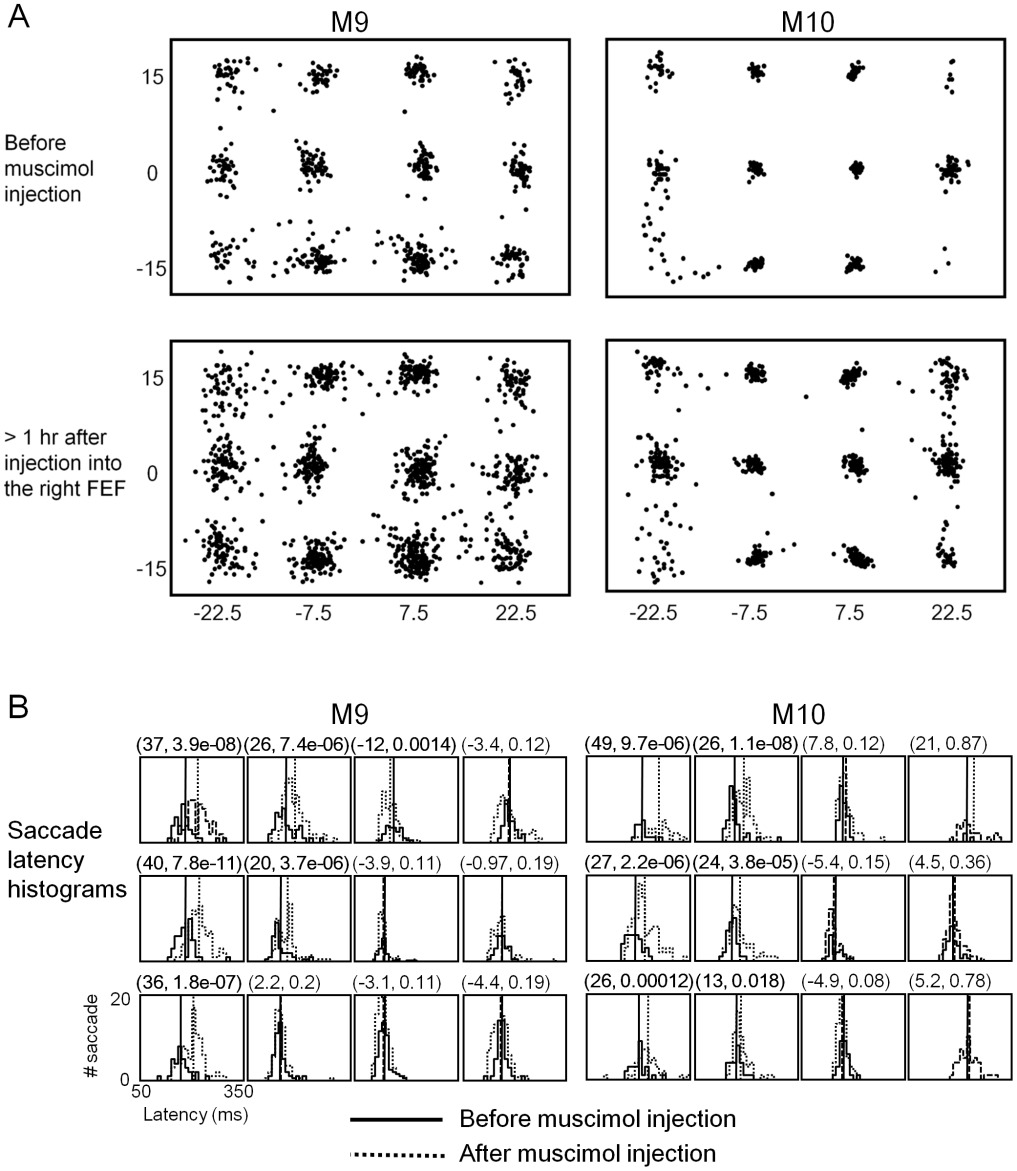
Minor effects on saccade parameters are observed during FEF inactivation. (**A**) The effect of FEF inactivation on the accuracy of saccades. End-points are shown of all saccades in three-target MEM sessions, before muscimol infusion into the right FEF (upper panels) and more than one hour after (lower panels). The post-muscimol data are pooled from multiple sessions to show approximately equal number of saccades to the contralesional locations. (**B**) FEF inactivation effect on saccade latency. The latency of the first, presumably visually-driven saccades are plotted in histograms, with the solid and broken curves representing data before and during inactivation, respectively. In each panel, the x axis indicates the latency in milliseconds, and the y axis the saccade count. Twelve panels in a set are arranged according to the target locations. The first number in a pair above each panel indicate the difference of median saccade latency (in ms) between pre- and post-injection data and the second the p value of the difference by the Kruskal-Wallis test. Significant differences (p<0.05) are printed in bold face. The latency increased by about 30 ms for contralesionally-directed visually-guided saccades during FEF inactivation.

**Figure 4:**
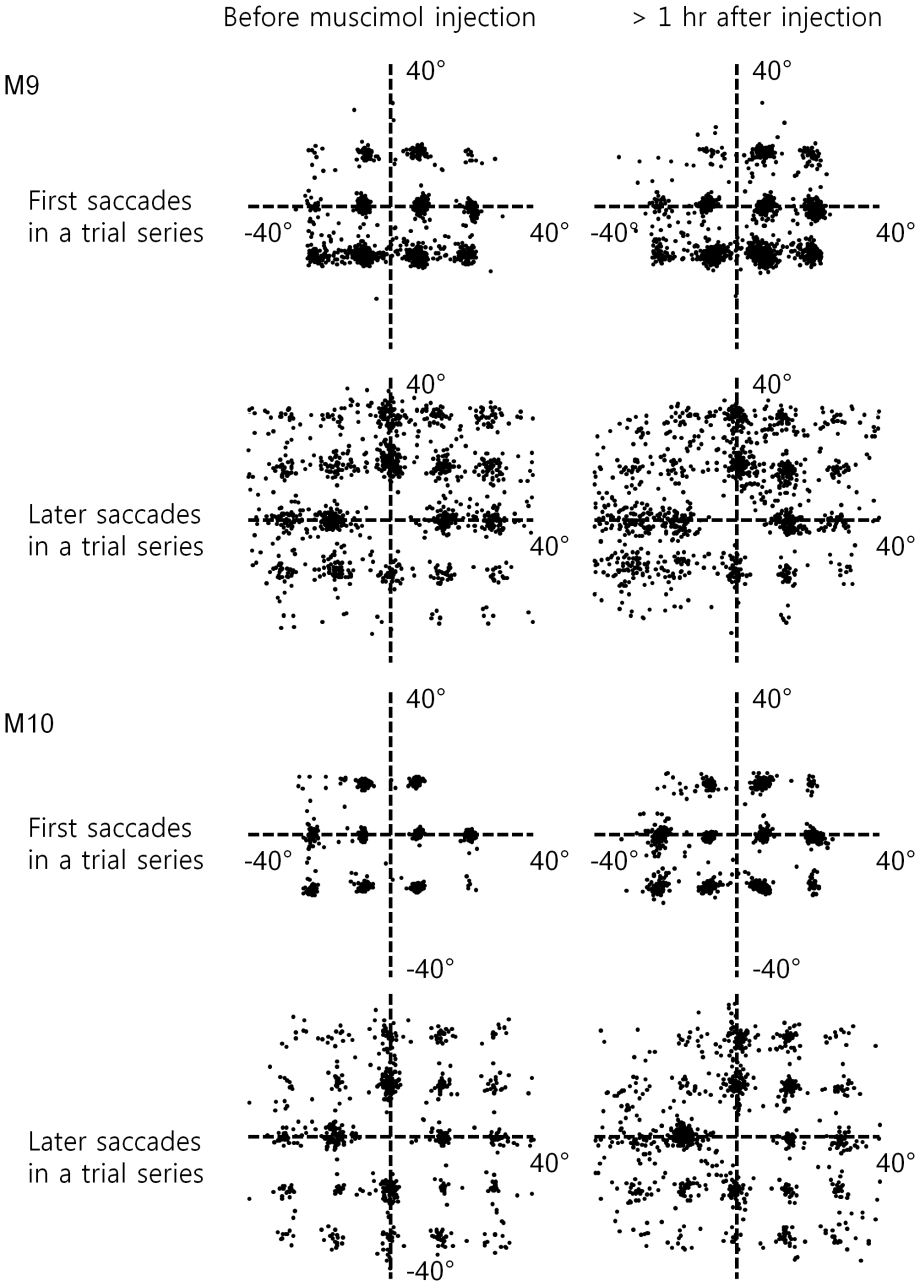
Saccade vectors for the three-target MEM task were comparable before and during FEF inactivation by muscimol. The first and later saccades of the saccade sequence in the trials were separately displayed. The data for M9 and M10 are shown in upper and lower sections, respectively. Both monkeys made downward saccades more often as the first saccade in the series. Note that later saccades driven by short-term memory tended to be multiples of 15 degrees in amplitude in both horizontal and vertical directions, which corresponded to the separation of discs in the matrix array. No obvious changes occurred during FEF inactivation, other than slight reduction in saccade frequency and increase in saccade vector variability when the saccade were directed contralesionally.

Minor changes in saccade latency and end-point accuracy were observed during FEF inactivation (Figures 3 and 4). There was an appreciable increase in dispersion of saccade endpoints around discs during the inactivation (Figure 3A), and the latency of first saccades in a series was delayed during inactivation by about 30 ms when they were directed contralesionally (Figure 3B). While consistent with previous observations [14], [15], [21], the slight increase in saccade inaccuracy and latency would not account for the decline in TSR in the MEM tasks, because the saccade dispersions were within the window boundaries set for detecting correctly targeted saccades (Figure 3A).

To delve into the reasons underlying the worse performance of MEM during FEF inactivation, saccade behavior was examined in detail. Specifically three aspects were considered as possible explanations for the TSR decline: overall frequency of generating saccades, relative frequency of saccades during rewarded and error trials, and target discriminability by individual saccades (Table 1).

**Table 1:** The FEF inactivation effect on the number of saccades per trial in the three-target memory task.

M9	Total saccades per trial	Contralesional saccades	Rewarded trials	On-target sac	1.41 → 1.15*
	2.97 → 2.59**	1.52 → 1.08**	1.63 → 1.36*	Not on-target	0.21 → 0.21
				TDI (%)	87.0 → 84.6
			Unrewarded trials	On-target sac	0.92 → 0.67*
			1.30 → 0.90*	Not on-target	0.38 → 0.23*
				TDI (%)	71.5 → 74.8
		Ipsilesional saccades	Rewarded trials	On-target sac	1.50 → 1.78*
		1.45 → 1.51	1.60 → 1.90*	Not on-target	0.10 → 0.12
				TDI (%)	93.8 → 93.8
			Unrewarded trials	On-target sac	0.94 → 1.12**
			1.10 → 1.28**	Not on-target	0.16 → 0.16
				TDI (%)	85.0 → 87.7
M10	Total saccades per trial	Contralesional saccades	Rewarded trials	On-target sac	1.46 → 1.32
	3.10 → 2.84*	1.54 → 1.31*	1.65 → 1.53	Not on-target	0.19 → 0.21
				TDI (%)	88.4 → 86.6
			Unrewarded trials	On-target sac	0.88 → 0.76
			1.11 → 0.99	Not on-target	0.22 → 0.23
				TDI (%)	80.6 → 76.9
		Ipsilesionalsaccades	Rewarded trials	On-target sac	1.49 → 1.64*
		1.57 → 1.53*	1.67 → 1.78*	Not on-target	0.18 → 0.14
				TDI (%)	89.4 → 92.1
			Unrewarded trials	On-target sac	1.06 → 1.08
			1.17 → 1.18	Not on-target	0.11 → 0.11
				TDI (%)	90.9 → 91.2

The values are the averages over five and six muscimol-injection experiments for M9 and M10, respectively. The first number in an arrowed pair is the saccade count before the inactivation and the second that during inactivation. Statistically significant differences between the two counts were marked by asterisks (*: p<0.05; **: p<0.01, by two-tailed paired t test). TDI: target discrimination index ( = on-target saccades/all saccades ×100).

First, we hypothesized that perhaps the animals became less prudent in saccade generation during FEF inactivation. Too many non-discriminatory saccades would have negatively affected the task performance, resulting in lower TSR. However, this hypothesis was not supported by the data: Both animals actually made significantly fewer saccades per trial when the FEF on one side was inactivated. As shown in the table, the total number of saccades per trial decreased by approximately 10%, in three-target MEM. The data also showed that the reduction was more prominent with saccades directed contralateral to the inactivated FEF for both animals (the second column in Table 1). Therefore, the decrease in TSR did not appear to result from less prudent saccade behavior, but rather from a reduction of contralesionally-directed saccades. Now, the reduction in saccade counts occurred only in situations requiring a high-load of short-term memory, i.e., in three-target MEM, and not in three-target VIS where the animals made as many saccades to both hemifields after muscimol injections as before them (saccades per trial in 3-target VIS: from 3.82 to 3.78 with muscimol injection in M9, p = 0.71, paired t-test; 3.63–>3.65 in M10, p = 0.56). Thus, it appeared that FEF inactivation specifically affected the memory of saccade targets, rather than visual or oculomotor aspects in saccade behavior.

Second, we compared the number of saccades between rewarded and unrewarded trials. If indiscriminate saccade behavior was responsible for the failure in unrewarded trails, the saccade count would be higher in these trials. To the contrary, there was fewer saccades in unrewarded in both animals, consistent with our impression that the animals were skilled enough on the tasks to refrain from making unnecessary saccades when unsure of where the targets had been. The animal apparently maintained this strategy during FEF inactivation, making fewer saccades in unrewarded trials. Presumably, the mnemonic representation of saccade targets was weakened by the inactivation.

Third, target discrimination by saccades was unaffected by FEF inactivation. As given in Table 1, there was no change in the target discrimination index (TDI), or the ratio of on-target saccades over all saccades. Even when the overall performance was impaired by FEF inactivation, the animals made saccades very selectively to the targets, and avoided non-targets as successfully as before the inactivation. Therefore, the decline in TSR was not attributable to indiscriminate saccade behavior. By the same token, increased scatter in saccade end-points during inactivation (Figure 3) could not account for the TSR decline either. Despite the saccade motor errors, TDI remained high (Table 1), i.e., saccades discriminated targets from non-targets very well during inactivation.

The above analyses on saccade behavior together made it rather unlikely that abnormalities in saccade execution could account for the impairment in MEM during FEF inactivation.

### The Impairment in MEM was Visual-field Specific and Load-dependent

To explore the relationship between spatial coding in the inactivated FEF sites and the memory impairment, we measured the saccade proportion to each target location, i.e., the proportion of trials in which at least one saccade was made to a location over all trials in which a target was shown at that location. Note that, per location, a target were shown N * m/12 times on average (N: the total number of trials, m: the target number on each trial), such that, if a total of 120 trials were administered, for each location, ten trials were expected to have a target at that location in the one-target VIS or MEM task, 20 trials in the two-target task, and so on. Therefore, the denominator of the saccade proportion varied depending on the target number in the tasks. On the other hand, the numerator in the saccade proportion was trials in which at least one saccade was made to a location on each trial. For this calculation, even if multiple saccades was made to a location on a single trial, the trial was counted only once. In this way, the saccade proportion was designed to quantify the saccade responses as a function of target location, normalizing the saccade behavior with respect to the target appearance in multi-target search and memory.

To evaluate the effect of FEF inactivation, the saccade proportion was compared before versus during FEF inactivation by a ratio (i.e., the saccade proportion ratio, SPR). Now, SPR close to one indicated no change in the saccade proportion, while zero meant complete disruption of memory saccades to a specific target location by the inactivation. (In theory, SPR can be higher than one, meaning better performance with FEF inactivation. However, no such case was observed in our experiments, likely because of the near-perfect performance before the inactivation.).

**Figure 5:**
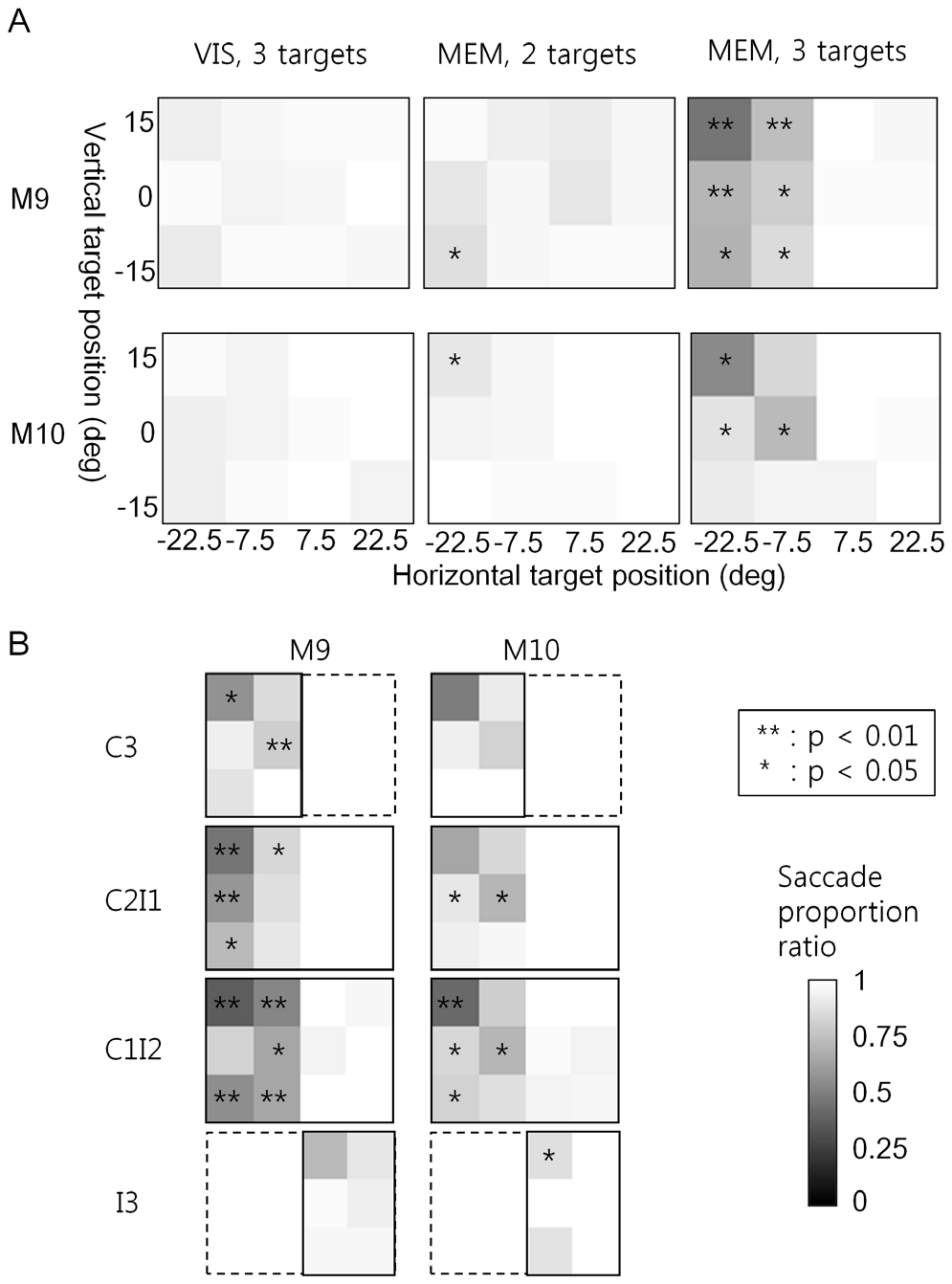
VSTM impairment during FEF inactivation was visual-field specific and dependent on target distribution. (**A**) Effects of FEF inactivation on the saccade behavior on each target location. The saccade proportion ratio (SPR) before and during inactivation is shown by grayscale images representing the layout of target locations. The saccade proportion is the proportion of trials in which a saccade was made to a target location over those where the target had appeared therein. Target locations in horizontal and vertical dimensions are marked in visual angle (degree). The saccade proportion decreased (SPR <1) during inactivation of the right FEF at target locations in the left upper visual fields, especially with the three-target memory (MEM) task. No decline of saccade proportion was observed with the multi-target search task (VIS). (**B**) FEF inactivation effects on the SPR as a function of the target distribution in the visual fields. The saccade proportion impairment was influenced by the layout of other accompanying targets in the three-target memory task. Trials were categorized into three groups by target distribution across the visual fields: C3 where all three targets were in the fields contralateral to muscimol infusion, C2I1 with two targets in contra- and one in ipsilateral fields, and so on. Visual fields in which the saccade proportion decreased during FEF inactivation were larger when more targets appeared in the ipsilateral fields (C3> C2I1> C1I2> I3). Asterisks indicate the p-values of paired t-tests comparing the saccade proportions before and during FEF inactivation: **, p<0.01; *, p<0.05. The grayscale bar is given at the bottom right, with the SPR ranging between zero and one.

In the grayscale images of Figure 5A, SPRs were shown in a 3×4 matrix corresponding to 12 target locations in the visual fields. No changes in the saccade proportion were observed (SPR of about one) for three-target VIS (the left column in Figure 5A).

In contrast, in the two- and three-target MEM (middle and right panels, respectively, in Figure 5A), the SPR was less than one, especially at location on the left-top quadrant, indicating that the saccade proportion decreased during FEF inactivation at these locations. This indicated that the impairment of overall memory performance (as indicated by the TSR data in Figure 2) resulted from the failure to make memory-guided saccades to these specific visual fields, which matched with the direction of electrically evoked saccades before muscimol infusion (Figure 1B).

### The Impairment of Multi-target Memory Depended on the Spatial Distribution of Targets

Given the spatially specific impairment in MEM, we next asked whether the memory failure was related to the deployment of visual attention at the time of target presentation. To investigate this possibility, we analyzed SPR as a function of spatial distribution of targets (Figure 5B). Trials were grouped according to the number of targets in the contra- or ipsi-lateral hemifields to the inactivated FEF: C3 (all three targets in the contralateral fields), C2I1 (two targets in contra- and one in ipsilateral fields), C1I2 (one contralateral and two ipsilateral targets), and I3 (all three targets in the ipsilateral fields). The rationale behind this grouping was that the accompanying targets would either boost or hinder attention to a target depending on where they were. For instance, a target in the inactivated field might be better remembered if other targets were on the same side, because attention drawn by these targets might enhance memory of the target. This was in fact what apparently happened in both animals: SPR at contralateral target locations (on the left side in each image in Figure 5B) decreased as a function of the number of targets in the opposite, ipsilateral hemi-field (C3> C2I1> C1I2). By the same token, the visual fields where the saccade proportion was negatively affected by FEF inactivation were larger, when more targets appeared in the ipsilateral fields (C3< C2I1< C1I2< I3). In the case of I3, the SPR decrease was observed even at the ipsilateral locations.

To check an alternative explanation for this target-distribution effect that the animals made more guessing saccades to the contralateral locations when targets had clustered on this side, the TDIs were compared across the trial groups. No significant difference was observed, regardless of the target distribution or the FEF inactivation.

## Discussion

### FEF Inactivation Reduced VSTM Capacity

FEF inactivation led to impairment of the multi-target memory-guided saccade task without significantly affecting serial saccades over the targets using salient color contrast. The impairment in MEM was load-dependent, and behaviorally speaking, the VSTM capacity was reduced by the inactivation. Based on the maximum number of targets that could be remembered in the MEM task, the VSTM capacity before FEF inactivation was estimated to be about three items. (This estimate included the first target which for sure was reached visually. However, whether this target entered and occupied a slot in the VSTM was not determinable in our experiments and tangential to the main findings in the study regarding the effect of FEF inactivation. Also, the estimate was obtained with the TSR of 50% regarded as the threshold. With a lower threshold, the estimate would be larger, without significantly altering our interpretations.) During the inactivation, the capacity was reduced to less than three in both animals: The performance in the three-target memory condition dropped to the chance level in M9, and to a significantly lower level in M10.

At the same time, the memory impairment was visual-field specific: it occurred only when a target was presented in the upper left visual fields, which matched with the direction of saccades electrically evoked at the inactivated FEF sites. Therefore, the FEF inactivation effect was conditional on both requirements, that is, high memory load and location of targets in the visual fields. Note in this regard that, while depicted in spatial coordinates of target locations, the memory deficit was in fact retinotopic since the animal fixated at the center when the targets were shown in the MEM task. The conjunction of visual-field specificity and memory-load dependence points to specific behavioral situations where the FEF are crucially required. This indicates that this cortical area is the neural structure where visual attention and VSTM interact with each other.

Only minor abnormalities in visually-guided saccades were observed during FEF inactivation previously (Figure 3) [13]–[16]. In keeping with this, normal performance was maintained in our multi-target search task (VIS in Figure 2), indicating that the inactivation did not significantly impinge on target detection and selection. On the other hand, serial saccades based on VSTM was impaired when the memory load approached the capacity limit. Given these findings, one may speculate that the size of neural population required for detecting and remembering the targets was different. Perhaps, a smaller population of FEF neurons would suffice for target detection than for short-term memory. Neurons that were relatively spared from inactivation could signal the targets for immediate saccades, but were unable to maintain the activity long enough over two saccades. Yet another possibility is that different neural elements are responsible for search versus memory: It is possible that visuo-movement neurons with sustained activity in the FEF [12] are crucial for the maintenance of target information, and inactivation of these neurons specifically would weaken the memory trace and hence impair the VSTM. Consistent with this notion of cell-type specific functions are the recent observations that shifts of gaze and shifts of attention may be carried out by different cell types [28] and even by different dopaminergic receptors [29] within this cortical area.

### Implications on FEF’s Role in Visual Attention and Short-term Memory

Current findings have a number of implications on the role played by the FEF in visual attention and VSTM [13]–[15], [22], [24], [30]. First, the fact that FEF inactivation did not affect the performance in visual search was consistent with the previous demonstration that posterior parietal neurons signaled the target location earlier than those in the frontal cortex in a visual search task similar to ours [22] and also in the change detection paradigm [31]. Given the selective impairment of VSTM, the FEF seemed more crucially involved in maintaining the saliency information after it was coded. In keeping with this distinction, a recent human fMRI study reported that the FEF, but not the parietal cortices, showed sustained delay-period activity for both the short-term memory and the attention tasks [32].

Second, our findings implicate the FEF as a neural substrate for the VSTM capacity. The inactivation effect on the MEM performance was load-dependent, which in behavioral terms amounted to a reduction in the VSTM capacity. Location memory has long been modeled as consisting of storage slots that are discrete in the sense that the entry into the slots is in an approximately all-or-none manner and does not critically depend on attentional effort during encoding [33], [34]. However, recently mounting evidence supports alternative views that the memory storage is not so discrete or fixed as previously assumed [35]–[37]. Whether entered into discrete slots [34] or encoded with variable precision [37], memory trace would first be established based on perceptual saliency which determines the priority of entry into the storage [38], and then sustained by FEF neurons.

The importance of the interaction between FEF and visual cortices for the VSTM capacity has been emphasized by a recent study where VSTM of location and object identity were investigated with functional MRI. FEF as well as parietal regions including the intraparietal sulcus exhibited activity related to the location VSTM [39]. Here, we demonstrated that FEF inactivation resulted in a reduction of VSTM capacity for target locations, and whether similar results will be obtained after inactivation of the parietal areas is certainly worth future investigation.

Third, the current results suggest that there is a dynamic competition during memory encoding of target locations, consistent with recent neurophysiological investigations using human [40] and non-human [31] subjects. In our study, the deficit in memory-guided saccades to the inactivated fields was exacerbated by accompanying targets in the opposite hemi-field, suggesting that concurrent neural activities encoding the targets inhibited one another. Furthermore, the competition was not confined to one FEF, but involved bilateral FEF’s at the same time. Cross-hemispheric interaction must be at play, given that activation of the intact FEF by a target(s) contralateral to the affected fields worsened the memory failure, whereas co-activation of the inactivated FEF by additional targets near the affected fields ameliorated it.

These considerations are consistent with the following neural model on how the FEF normally functions in visual attention and VSTM: Being spatially coded and provided with visual signals from posterior cortices, FEF neurons may determine the entry of visual information into storage. As targets and non-targets are distinguished by color contrast, ensembles of FEF neurons get activated by neurons at more posterior parts of the brain, and a competition will start among the ensembles. The entry into VSTM storage will then depend on whether an ensemble grows beyond a size large enough to establish a self-sustaining activation. Important insights from our study are 1) that the size of FEF matters in this process: That is, the cortical area can concurrently support only a limited number of such large ensembles, and the entry into VSTM is allowed only up to this number. 2) Our data also suggest that the neuronal ensembles encoding saccade targets compete to recruit from the limited population of FEF neurons. The extent and the activity level of the competing ensembles of active neurons might represent the averaged sum of discrete resources assigned to slots in the slots-plus-averaging model [34] or correspond to the mnemonic representation of stimuli with variable gain and precision in the variable-precision model [37]. When a chemical lesion renders part of the FEF unexcitable, the number of self-sustainable ensembles will further decline resulting in a reduction of VSTM capacity. In this sense, the VSTM capacity is a behavioral manifestation of the limited expanse of neural tissue in FEF.

### Limits of Our Experiments in Investigating VSTM

Our results alone could not determine whether the effect of FEF inactivation was on the memory trace *per se* or on updating the memory after saccades [41], [42]. With this distinction in mind, we analyzed error trials and compared TSR as a function of the first-saccade direction: If our monkeys had behaved like a human patient with a right frontoparietal lesion who was impaired in double-step saccades only when the first saccade was contralesionally directed [43], the idea would be supported that FEF inactivation disrupted space remapping after the saccade by corollary discharge. However, there was no difference in TSR regardless of the direction of the first saccades: In the three-target memory condition and two-hours after muscimol injection, TSR of M9 were 0.36 and 0.37 with the first saccade directed contralesionally and ipsilesionally, respectively (p = 0.65, two-tailed paired t-test). Likewise, TSR of M10 was 0.61 and 0.57 (p = 0.27). Moreover, given the preserved performance in the two-target memory condition, it was clear that the mnemonic representation of the target for the second saccades was normally updated after the first saccades despite FEF inactivation, at least under low memory-load. Obviously, this finding does not necessarily rule out the FEF’s role in saccade remapping: In fact, the muscimol effect might have accumulated over a sequence of saccade and hence the deficit was seen only with high memory-load. Given that human patients with remapping impairments between saccades had lesions involving the parietal lobe [43], [44], inactivation experiments on the parietal oculomotor areas in monkeys might prove more elucidating in this regard.

Neither was determinable by our experiments whether mnemonic representations of the saccade target were rendered weaker in strength or fewer in number by FEF inactivation. Behaviorally, either situation would result in the same TSR decline, because we probed the memory trace by saccade responses and consequently the probing was stretched over time. With more targets to remember, the time taken by the saccade series also lengthened. Therefore, we cannot tell whether FEF inactivation have affected the memory by limiting the number of targets encoded, or by accelerating the memory decay over time.

An issue may be raised regarding the short and variable retention times in our memory task: Saccades were made immediately after visual encoding of the targets and the retention time for each target was variable because the saccade responses were made in sequence. Thus, our task might have tapped on the iconic memory [45], and the mnemonic representations of saccade targets might not have been stabilized when the saccade responses were triggered. This would not, however, invalidate our view that the deployment of spatial attention, as enacted by FEF, plays a pivotal role in the stabilization (i.e., encoding) process. In our opinion, whether the visual short-term memory capacity is imposed at the encoding stage or during stable retention is of some theoretical importance but probably indistinguishable in neural terms, given the highly dynamic nature of neural activity underlying the mnemonic representations [31].

## References

[1] Marois R, Ivanoff J (2005) Capacity limits of information processing in the brain. Trends Cogn Sci 9: 296–305. 15925809 10.1016/j.tics.2005.04.010

[2] Wright AA (2007) An experimental analysis of memory processing. J Exp Anal Behav 88: 405–433. 18047230 10.1901/jeab.2007.88-405 PMC2174369

[3] Heyselaar E, Johnston K, Paré M (2011) A change detection approach to study visual working memory of the macaque monkey. J Vis 11: 11. 10.1167/11.3.11 21402883

[4] Todd JJ, Marois R (2004) Capacity limit of visual short-term memory in human posterior parietal cortex. Nature 428: 751–754. 15085133 10.1038/nature02466

[5] Vogel EK, Machizawa MG (2004) Neural activity predicts individual differences in visual working memory capacity. Nature 428: 748–751. 15085132 10.1038/nature02447

[6] Luck SJ, Vogel EK (1997) The capacity of visual working memory for features and conjunctions. Nature 390: 279–281. 9384378 10.1038/36846

[7] Cowan N (2001) The magical number 4 in short-term memory: a reconsideration of mental storage capacity. Behav Brain Sci 24: 87–114; discussion 114–185. 10.1017/s0140525x01003922 11515286

[8] Wheeler ME, Treisman AM (2002) Binding in short-term visual memory. J Exp Psychol Gen 131: 48–64. 11900102 10.1037//0096-3445.131.1.48

[9] Elmore LC, Ma WJ, Magnotti JF, Leising KJ, Passaro AD, et al (2011) Visual short-term memory compared in rhesus monkeys and humans. Curr Biol 21: 975–979. 21596568 10.1016/j.cub.2011.04.031 PMC4634532

[10] Lara AH, Wallis JD (2012) Capacity and precision in an animal model of visual short-term memory. J Vis 12. 10.1167/12.3.13 PMC3549639 22419756

[11] Inoue M, Mikami A, Ando I, Tsukada H (2004) Functional brain mapping of the macaque related to spatial working memory as revealed by PET. Cereb Cortex 14: 106–119. 14654462 10.1093/cercor/bhg109

[12] Bruce CJ, Goldberg ME (1985) Primate frontal eye fields. I. Single neurons discharging before saccades. Journal of Neurophysiology 53: 603–635. 3981231 10.1152/jn.1985.53.3.603

[13] Dias EC, Kiesau M, Segraves MA (1995) Acute activation and inactivation of macaque frontal eye field with GABA-related drugs. Journal of Neurophysiology 74: 2744–2748. 8747229 10.1152/jn.1995.74.6.2744

[14] Sommer MA, Tehovnik EJ (1997) Reversible inactivation of macaque frontal eye field. Exp Brain Res 116: 229–249. 9348123 10.1007/pl00005752

[15] Dias EC, Segraves MA (1999) Muscimol-induced inactivation of monkey frontal eye field: effects on visually and memory-guided saccades. J Neurophysiol 81: 2191–2214. 10322059 10.1152/jn.1999.81.5.2191

[16] Keller EL, Lee KM, Park SW, Hill JA (2008) Effect of inactivation of the cortical frontal eye field on saccades generated in a choice response paradigm. J Neurophysiol 100: 2726–2737. 18784274 10.1152/jn.90673.2008 PMC2585392

[17] Alvarez GA, Cavanagh P (2005) Independent resources for attentional tracking in the left and right visual hemifields. Psychol Sci 16: 637–643. 16102067 10.1111/j.1467-9280.2005.01587.x

[18] Umemoto A, Drew T, Ester EF, Awh E (2010) A bilateral advantage for storage in visual working memory. Cognition 117: 69–79. 20659731 10.1016/j.cognition.2010.07.001 PMC2934872

[19] Alvarez GA, Gill J, Cavanagh P (2012) Anatomical constraints on attention: hemifield independence is a signature of multifocal spatial selection. J Vis 12: 9. 10.1167/12.5.9 PMC4498678 22637710

[20] Schall JD, Hanes DP (1993) Neural basis of saccade target selection in frontal eye field during visual search. Nature 366: 467–469. 8247155 10.1038/366467a0

[21] Wardak C, Ibos G, Duhamel JR, Olivier E (2006) Contribution of the monkey frontal eye field to covert visual attention. J Neurosci 26: 4228–4235. 16624943 10.1523/JNEUROSCI.3336-05.2006 PMC6674003

[22] Buschman TJ, Miller EK (2007) Top-down versus bottom-up control of attention in the prefrontal and posterior parietal cortices. Science 315: 1860–1862. 17395832 10.1126/science.1138071

[23] Monosov IE, Thompson KG (2009) Frontal eye field activity enhances object identification during covert visual search. J Neurophysiol 102: 3656–3672. 19828723 10.1152/jn.00750.2009 PMC2804410

[24] Buschman TJ, Miller EK (2009) Serial, covert shifts of attention during visual search are reflected by the frontal eye fields and correlated with population oscillations. Neuron 63: 386–396. 19679077 10.1016/j.neuron.2009.06.020 PMC2758537

[25] Wardak C, Vanduffel W, Orban GA (2010) Searching for a salient target involves frontal regions. Cereb Cortex 20: 2464–2477. 20100901 10.1093/cercor/bhp315

[26] AVMA Panel on Euthanasia (2001) 2000 Report of the AVMA Panel on Euthanasia. J Am Vet Med Assoc 218: 669–696. 11280396 10.2460/javma.2001.218.669

[27] Lee KM, Ahn KH, Keller EL (2012) Saccade generation by the frontal eye fields in rhesus monkeys is separable from visual detection and bottom-up attention shift. PLoS One 7: e39886. 22761923 10.1371/journal.pone.0039886 PMC3384609

[28] Gregoriou GG, Gotts SJ, Desimone R (2012) Cell-type-specific synchronization of neural activity in FEF with V4 during attention. Neuron 73: 581–594. 22325208 10.1016/j.neuron.2011.12.019 PMC3297082

[29] Noudoost B, Moore T (2011) Control of visual cortical signals by prefrontal dopamine. Nature 474: 372–375. 21572439 10.1038/nature09995 PMC3117113

[30] Moore T, Fallah M (2004) Microstimulation of the frontal eye field and its effects on covert spatial attention. J Neurophysiol 91: 152–162. 13679398 10.1152/jn.00741.2002

[31] Buschman TJ, Siegel M, Roy JE, Miller EK (2011) Neural substrates of cognitive capacity limitations. Proc Natl Acad Sci U S A 108: 11252–11255. 21690375 10.1073/pnas.1104666108 PMC3131328

[32] Offen S, Gardner JL, Schluppeck D, Heeger DJ (2010) Differential roles for frontal eye fields (FEFs) and intraparietal sulcus (IPS) in visual working memory and visual attention. J Vis 10: 28. 10.1167/10.11.28 PMC2989410 20884523

[33] Rouder JN, Morey RD, Cowan N, Zwilling CE, Morey CC, et al (2008) An assessment of fixed-capacity models of visual working memory. Proc Natl Acad Sci U S A 105: 5975–5979. 18420818 10.1073/pnas.0711295105 PMC2329704

[34] Zhang W, Luck SJ (2008) Discrete fixed-resolution representations in visual working memory. Nature 453: 233–235. 18385672 10.1038/nature06860 PMC2588137

[35] Bays PM, Husain M (2008) Dynamic shifts of limited working memory resources in human vision. Science 321: 851–854. 18687968 10.1126/science.1158023 PMC2532743

[36] Cowan N, Rouder JN (2009) Comment on “Dynamic shifts of limited working memory resources in human vision”. Science 323: 877; author reply 877. 10.1126/science.1166478 PMC2730043 19213899

[37] van den Berg R, Shin H, Chou WC, George R, Ma WJ (2012) Variability in encoding precision accounts for visual short-term memory limitations. Proc Natl Acad Sci U S A 109: 8780–8785. 22582168 10.1073/pnas.1117465109 PMC3365149

[38] Spence C, Parise C (2010) Prior-entry: a review. Conscious Cogn 19: 364–379. 20056554 10.1016/j.concog.2009.12.001

[39] Harrison A, Jolicoeur P, Marois R (2010) “What” and “where” in the intraparietal sulcus: an FMRI study of object identity and location in visual short-term memory. Cereb Cortex 20: 2478–2485. 20100899 10.1093/cercor/bhp314 PMC2936801

[40] Vogel EK, McCollough AW, Machizawa MG (2005) Neural measures reveal individual differences in controlling access to working memory. Nature 438: 500–503. 16306992 10.1038/nature04171

[41] Colby CL, Goldberg ME (1999) Space and attention in parietal cortex. Annu Rev Neurosci 22: 319–349. 10202542 10.1146/annurev.neuro.22.1.319

[42] Bays PM, Husain M (2007) Spatial remapping of the visual world across saccades. Neuroreport 18: 1207–1213. 17632269 10.1097/WNR.0b013e328244e6c3 PMC2531238

[43] Duhamel JR, Goldberg ME, Fitzgibbon EJ, Sirigu A, Grafman J (1992) Saccadic dysmetria in a patient with a right frontoparietal lesion. The importance of corollary discharge for accurate spatial behaviour. Brain 115 (Pt 5): 1387–1402. 10.1093/brain/115.5.1387 1422794

[44] Pisella L, Mattingley JB (2004) The contribution of spatial remapping impairments to unilateral visual neglect. Neurosci Biobehav Rev 28: 181–200. 15172763 10.1016/j.neubiorev.2004.03.003

[45] Lu ZL, Neuse J, Madigan S, Dosher BA (2005) Fast decay of iconic memory in observers with mild cognitive impairments. Proc Natl Acad Sci U S A 102: 1797–1802. 15665101 10.1073/pnas.0408402102 PMC547847

